# Anti-tumor mechanism of artesunate

**DOI:** 10.3389/fphar.2024.1483049

**Published:** 2024-10-25

**Authors:** Xiaoyu Fan, Yiming Yan, Yafei Li, Yu Song, Bo Li

**Affiliations:** Department of Oral Anatomy and Physiology, Jilin Provincial Key Laboratory of Oral Biomedical Engineering, Hospital of Stomatology, Jilin University, Changchun, China

**Keywords:** artesunate, anti-tumor, mechanism, cancer, drug

## Abstract

Artesunate (ART) is a classic antimalarial drug with high efficiency, low toxicity and tolerance. It has been shown to be safe and has good anti-tumor effect. Existing clinical studies have shown that the anti-tumor mechanisms of ART mainly include inducing apoptosis and autophagy of tumor cells, affecting tumor microenvironment, regulating immune response, overcoming drug resistance, as well as inhibiting tumor cell proliferation, migration, invasion, and angiogenesis. ART has been proven to fight against lung cancer, hepatocarcinoma, lymphoma, multiple myeloma, leukemia, colorectal cancer, ovarian cancer, cervical cancer, malignant melanoma, oral squamous cell carcinoma, bladder cancer, prostate cancer and other neoplasms. In this review, we highlight the effects of ART on various tumors with an emphasis on its anti-tumor mechanism, which is helpful to propose the potential research directions of ART and expand its clinical application.

## Graphical Abstract

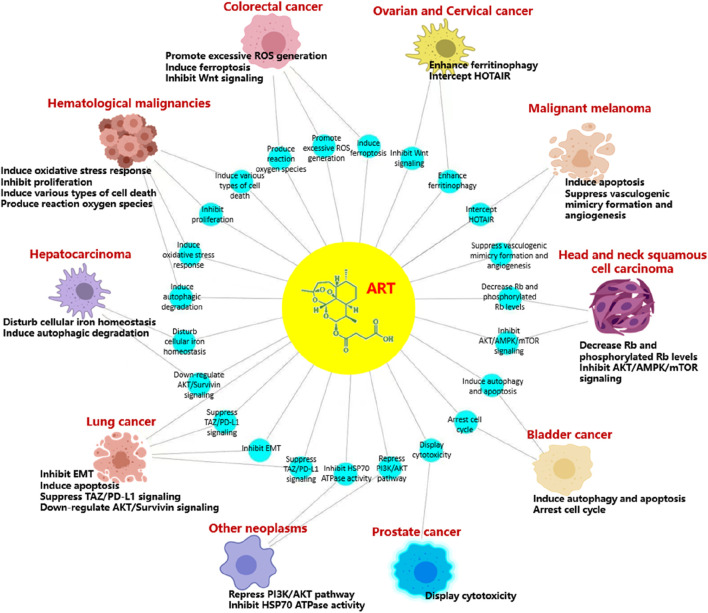

## 1 Introduction

Chemoresistance is an important cause of tumor treatment failure and recurrence. Therefore, it is of great significance to find effective anti-tumor drugs that can overcome drug resistance. Artesunate (ART), an efficient and relatively safe water-soluble hemisuccinate derivative of dihydroartemisinin (DHA) (an antimalarial drug) (Qian Y. et al., 2021), is derived from artemisia annua. ART is inherently unstable in an aqueous solution, rapidly hydrolyzes after preparation and injection, and can be used for temporary intravenous administration (Gashe et al., 2022). At present, it has been found that two-carbon tied artemisinin-isatin complexes have anti-breast cancer potential and the artemisinin-isatin hybrid with ethylene binding system has anti-lung cancer activity (Wang et al., 2023a; Wang et al., 2023b). ART is inherently unstable in an aqueous solution, rapidly hydrolyzes after preparation and injection, and can be used for temporary intravenous administration. Recent studies have found that in addition to its specific anti-malaria effect, ART also has many other biological activities, such as anti-inflammatory (Ruwizhi et al., 2022), anti-tumor (Eitae and Park, 2020), anti-viral (Hu Y. J. et al., 2021) and hypoglycemic (Alagbonsi et al., 2021). Especially in the field of anti-tumor, several studies have reported the strong anticancer activity of ART in recent years (Ma et al., 2021; Guan et al., 2023; Zeng et al., 2023). The anti-tumor mechanism of ART is largely dependent on the release of DHA from ART as a prodrug. The latest research shows that DHA inhibits tumor progress via blocking ROR1-induced STAT3-activation (Li J.-Y et al., 2022; Li et al., 2024b), inhibits angiogenesis via regulating VEGF and MMP-2/-9 (Rao et al., 2024), remodels tumor micro-environment and improves cancer immunotherapy through inhibiting cyclin-dependent kinases (Zhou et al., 2024), restores the immunogenicity and enhances the anticancer immunosurveillance of cisplatin by activating the PERK/eIF_2_α pathway (Li G et al., 2022). The other potential anti-tumor mechanisms of ART may be its own mechanism of action. ART inhibits the proliferation of Burkitt lymphoma cells by inhibiting AKT and ERK, so this compound is considered to have the potential to develop novel anti-tumor drugs (Yuan-Ce et al., 2023). Related studies have shown that ART acts on glioblastoma cells by inducing oxidative stress, DNA damage, apoptosis and necrosis (Strik et al., 2024). ART can also induce apoptosis of breast cancer cells, showing an anti-breast cancer effect (Wen et al., 2024). ART could combat choroidal melanoma by promoting apoptosis, inducing cell cycle arrest, and increasing intracellular ROS levels (Ma et al., 2024a). The review aims to exhibit the different mechanisms of ART against tumors, such as inhibiting proliferation and inducing apoptosis, and the latest research progress in different tumors elucidating the anti-tumor mechanism of ART, and lay a theoretical foundation for further development of ART therapy for cancer.

## 2 Discovery of ART and its clinical application

Artemisia annua is an anti-fever herbal medicine first described in traditional Chinese medicine 1,000 years ago. Artemisinin (ARS), the extract of Artemisia annua, and its derivatives (DHA, artemether, and ART) have been used for the treatment of malaria with substantial efficacy (Zhang T. et al., 2020). As a classic antimalarial drug, ART is widely used to treat malaria caused by the multidrug-resistant strains of *Plasmodium falciparum* (Adebayo et al., 2020) for its advantages of high potency, low toxicity (Shi et al., 2022), hydrophilic properties, better bioavailability than ARS (Markowitsch et al., 2020), and good tolerance without side effects (Mancuso et al., 2021b), which has broad development prospects. According to the results of relevant experiments, ART inhibits the growth and survival of many types of cancer including leukemia, melanoma cancer, bladder cancer, ovarian cancer, cervical cancer, breast cancer, colon cancer, head and neck tumors, hepatocellular carcinoma, prostate cancer, and multiple myeloma (Hao et al., 2020; Ma and Fei, 2020; Wen et al., 2024), etc. The role of ART against different cancers along with cell proliferation, apoptosis, western and IC50 can be abserved from Table 1. For example, artesunate induces a decrease in Rb and phosphorylated Rb levels and thus promotes Head and neck squamous cell carcinoma (HNSCC) cell apoptosis (Okamoto et al., 2023). ART induces autophagy of tumor cells by activating AMPK-mTOR axis, which plays an anti-glioblastoma multiforme (GBM) role (Ding et al., 2023). Recent studies on the anti-tumor mechanism of ART have shown that ART produces ROS (Zhao P. H. et al., 2020) and causes apoptosis of cancer cells (Yin et al., 2020). Another study reveals that ART elevates the apoptotic rate and suppresses C918 cell (choroidal melanoma cells of the human eye) viability by regulating the MALAT1/YAP signaling pathway (Jiu et al., 2021). In addition, ART inhibits tumor invasion and metastasis by inhibiting angiogenesis and vasculogenic mimicry (VM) formation (Geng et al., 2022). ART has been shown to be safe (Augustin et al., 2020) and effective for clinical use, moreover, it has a wider range of potential clinical applications than originally hypothesized because of its greater biological activity (Yang et al., 2021).

**TABLE 1:** The role of ART against different cancer along with other activities (cell proliferation/apoptosis/Western blot/IC50).

Neoplasms	Proliferation, invasion and metastasis	Apoptosis and autophagy	Western blot	IC_50_ (μM)	References
Lung cancer	Inhibit EMT, downregulate the transcription of MMP-2 and MMP-7	Regulate mitochondrial membrane potential	Bcl-2 Bax	H1975:4.023 LLC:11.05 H468:16.11	Wang et al. (2020a), Zhao et al. (2020c), Yang et al. (2021), Cao et al. (2022)
Hepatocarcinoma	Alter the expression and activity of regulatory enzymes in the cell cycle	Target GBA, increase intracellular ROS levels	Bcl-2 Bax	HepG2:38.38 MHCC-97H:171.4	Yin et al. (2020), Chen et al. (2022)
Lymphoma	Combine with Hsp90 to reduce the expression of AKT, ERK, p-AKT, p-ERK, and EGFR	Induce ferroptosis by regulating metallothionein 1G, induce apoptosis via a caspase-dependent pathway	caspase-3 caspase-8 caspase-9 PARP Bcl-2 IAP	SU-DHL-4:0.89 ± 0.12 Daudi:0.70 ± 0.29 CA-46:0.72 ± 0.05 JEKO-1:1.34 ± 0.31	Ishikawa and Mori (2021), Yuan-Ce et al. (2023), Xiong et al. (2024)
Myeloma	Arrest the cell cycle at G0/G1 phase with downregulation of cyclin D, CDK2, and CDK4	Induce ferroptosis by inhibiting the nuclear localization of Sterol regulatory element binding protein 2 (SREBP2)	GPX4 ACSL4	MM1S:53.61 RPMI8226:58.96	Mancuso et al. (2021b), Liang et al. (2023)
Leukemia	Downregulate SRC, downregulate levels of activator protein-1 (AP-1) and NF-κB signaling	Induce apoptosis through the mitochondrial pathway via generation of ROS	JNK caspase-3 caspase-7 caspase-8 Bcl-2	MV4-11:0.2529 THP-1:0.3664	Mancuso et al. (2021b), Hill et al. (2023)
Ovarian cancer	Arrest the cell cycle at the G0/G1 phase	Trigger the intrinsic apoptotic cascade involving cytochrome c, elevate the intracellular levels of ROS	Bcl-2 caspase-3 P53	OVCAR3:5.95 UWB1.289:17.95 CAOV3:26.73 OV-90:61.00	McDowell et al. (2021a), Li et al. (2022a), McCorkle et al. (2024)
Bladder cancer	Inhibit cellular lipogenesis associated with the Clusterin/SREBP1/FASN signaling pathway	Upregulate ROS, activate AMPK-mTOR-ULK1 axis	Bax caspase-3 caspase-PARP Bcl-2	EJ: 89 T24: 95	Zhou et al. (2020a), Lin et al. (2024a)
Prostate cancer	Arrest the cell cycle at the G0/G1 phase	Trigger ferroptosis by consuming extracellular GSH, decreasing GPX4 levels and generating ROS (Yu et al.; Xiao et al., 2020; Okamoto et al., 2023)	caspase 3 PARP-1	PC-3:25.1 LnCaP:2.13	Fabian et al. (2022), Vakhrusheva et al. (2022)
Breast cancer	Block cell cycle in G2/M (ROS-dependent) and in G1 (ROS-independent)	Decrease expression and activity of HSP70, influence the transcripts for Eph receptors and ephrin ligands	HSP70 Bcl-2 caspase-9	MCF-7:83.28 4T1:52.41	Duarte and Vale (2020), Zadeh et al. (2022)

## 3 Antineoplastic mechanism of ART

The main mechanism of ARS and its derivatives (ART, artemether, DHA) toward leukemia, multiple myeloma, and lymphoma cells comprises oxidative stress response, inhibition of proliferation, induction of various types of cell death as apoptosis, autophagy, ferroptosis, inhibition of angiogenesis, and signal transducers, such as NF-κB, MYC, amongst others (Mancuso et al., 2021b). Existing basic and clinical studies have shown that the pharmacological mechanism of ART therapy for cancer is embodied in the inhibition of tumor cell proliferation, migration and invasion (Xu et al., 2022), induction of tumor cell apoptosis and autophagy (Zhao F. G. et al., 2020), regulation of cell signal transduction (Zeng et al., 2022), inhibition of tumor angiogenesis (Lu et al., 2022b) and others (Table 2).

**TABLE 2:** Summary of antineoplastic mechanism and signaling pathway of artesunate.

Mechanism of action	Signaling pathway	References
Inhibition of tumor cell proliferation, invasion and metastasis	AMPK pathway, Akt/mTOR pathway, PI3K/AKT/FKHR pathway, MEK/ERK pathway, ERK/c-Myc pathway, STAT3 pathway, Wnt pathway	Antoszczak et al. (2020), Chen et al. (2020), Wang et al. (2020a), Xiao et al. (2020), Yin et al. (2020), Berkoz et al. (2021), Hamoya et al. (2021), Li et al. (2021a), Hu et al. (2022), Huang et al. (2022a), Xu et al. (2022), Zhang et al. (2022e), Liu et al. (2023a)
Induction of tumor cell apoptosis and autophagy	STAT3 signaling pathway, AMPK-mTOR-ULK1 axis	Zhou et al. (2020b), Chen et al. (2021)
Inhibition of tumor angiogenesis	STAT3 signaling pathway HIF-1α/VEGF/PDGF pathway STAT3/AKT signaling pathway	Berkoz et al. (2021), Chen et al. (2023b) Ma et al. (2024b) Yao et al. (2024b)
Affecting the tumor microenvironment	JAK2/STAT3 signaling pathway	Mancuso et al. (2021c)
Modulating immune response	TAZ/PD-L1 signaling pathway	Cao et al. (2022)
Overcoming drug resistance in cancer cells	TAZ signaling pathway JAK/STAT3 signaling pathway Noxa/Bim/Mcl-1/p-Chk1 axis AFAP1L2-SRC-FUNDC1 axis-dependent mitochondrial autophagy	Cao et al. (2022) Su et al. (2023) Zhang et al. (2022a) Ma et al. (2024c)

### 3.1 Inhibition of tumor cell proliferation, invasion and metastasis

Studies have shown that ART can inhibit the proliferation of tumor cells by inhibiting or activating certain signal pathways (Antoszczak et al., 2020). ART causes mitochondrial dysfunction to further activate AMPK and suppress Akt/mTOR (Xiao et al., 2020). ART induces apoptosis in thyroid cancer cells and inhibits their proliferation and migration by inhibiting the PI3K/AKT/FKHR signal pathway (Xu et al., 2022). ART induces apoptosis of leukemia cells and inhibits the growth and stemness of transplanted tumors via the suppression of the MEK/ERK and PI3K/Akt pathways (Chen et al., 2020). ART evidently attenuates the migration, invasion and proliferation of cutaneous squamous cell carcinoma (CSCC) cells, which may be intensely related to PI3K/AKT pathway repression (Huang X. Y. et al., 2022). Another study proves that DHA and ART inhibit the growth of non-small cell lung cancer (NSCLC) via prohibiting cancer cell aerobic glycolysis through ERK/c-Myc pathway (Zhang Y. X. et al., 2022). ART can also inhibit cell proliferation, invasion and metastasis by affecting protein and enzyme expression. For instance, ART induces a significant downregulation of cyclin-dependent kinase-2 (CDK2), CDK4, cyclin D1, and cyclin E1 at various levels and then causes apoptosis, which impairs normal liver cell proliferation by inducing G0/G1 cell cycle arrest and apoptosis (Yin et al., 2020). ART combined with cisplatin (CIS) exerts anticancer effects on A549 cells by influencing the expression of Bcl-2, Box, p-P53, Caspase-3/7, Caspase-9, Cyclin Bl, P34, P21 (Li W. et al., 2021; Liu W. et al., 2023). The antimigration activity of ART is mediated by inhibition of BTBD7 mRNA expression while BTBD7 was found highly expressed in tumor tissues of NSCLC patients (Wang J. S. et al., 2020). ART may suppress the proliferation, migration and invasion of A549 and H1299 cells and induce their apoptosis by decreasing the expressions of human antigen R and matrix metalloproteinase-9 (MMP-9) proteins (Hu et al., 2022). The results obtained from another study demonstrate that the anticancer activity of ART occurs via STAT3 pathway and its target proteins (Berkoz et al., 2021). In addition, ART inhibits intestinal tumorigenesis by inhibiting Wnt signal pathway (Hamoya et al., 2021).

### 3.2 Induction of tumor cell apoptosis and autophagy

ART evokes ferroptosis, an iron-dependent cell death caused by ROS formation (Olivo et al., 2022; Huang et al., 2023). Accordingly, ferroptotic effects have been demonstrated in ART-induced head and neck tumor cells, pancreatic cancer cells, liver cancer cells (Markowitsch et al., 2020), ovarian serous carcinoma cells (Koike et al., 2022) and glioblastoma cells (Song et al., 2022). ARS derivatives have been shown to have anti-NSCLC activity through induction of ROS-dependent apoptosis/ferroptosis (Zhang Q. T. et al., 2021). ART may induce apoptosis and cell cycle arrest to inhibit cell proliferation, and regulate autophagy and ferroptosis via impairing the STAT3 signaling pathway in diffuse large B cell lymphoma (DLBCL) cells (Chen et al., 2021). ART targets oral tongue squamous cell carcinoma via mitochondrial dysfunction-dependent oxidative damage (Xiao et al., 2020). ART treatment causes significant growth inhibition and apoptosis in A549 cells (Zhang W. W. et al., 2022) and induces apoptosis in breast cancer cells as a HSP70 ATPase activity inhibitor (Pirali et al., 2020). ART has an anti-esophageal cancer effect by inhibiting aerobic glycolysis (Jin et al., 2022). Furthermore, ART induces autophagy dependent apoptosis through upregulating ROS and activating AMPK-mTOR-ULK1 axis in human bladder cancer cells (Zhou X. J. et al., 2020). The researchers demonstrate that high GBA levels over activated autophagic flux, accelerates the rate at which cellular material may be degraded and recycled in balanced, healthy cells. This disturbance enables liver cancer to progress while ART can suppress GBA expression levels and restore normal autophagic flux, boosting the drug’s anticancer activity (Chen et al., 2022).

### 3.3 Inhibition of tumor angiogenesis

**FIGURE 1:**
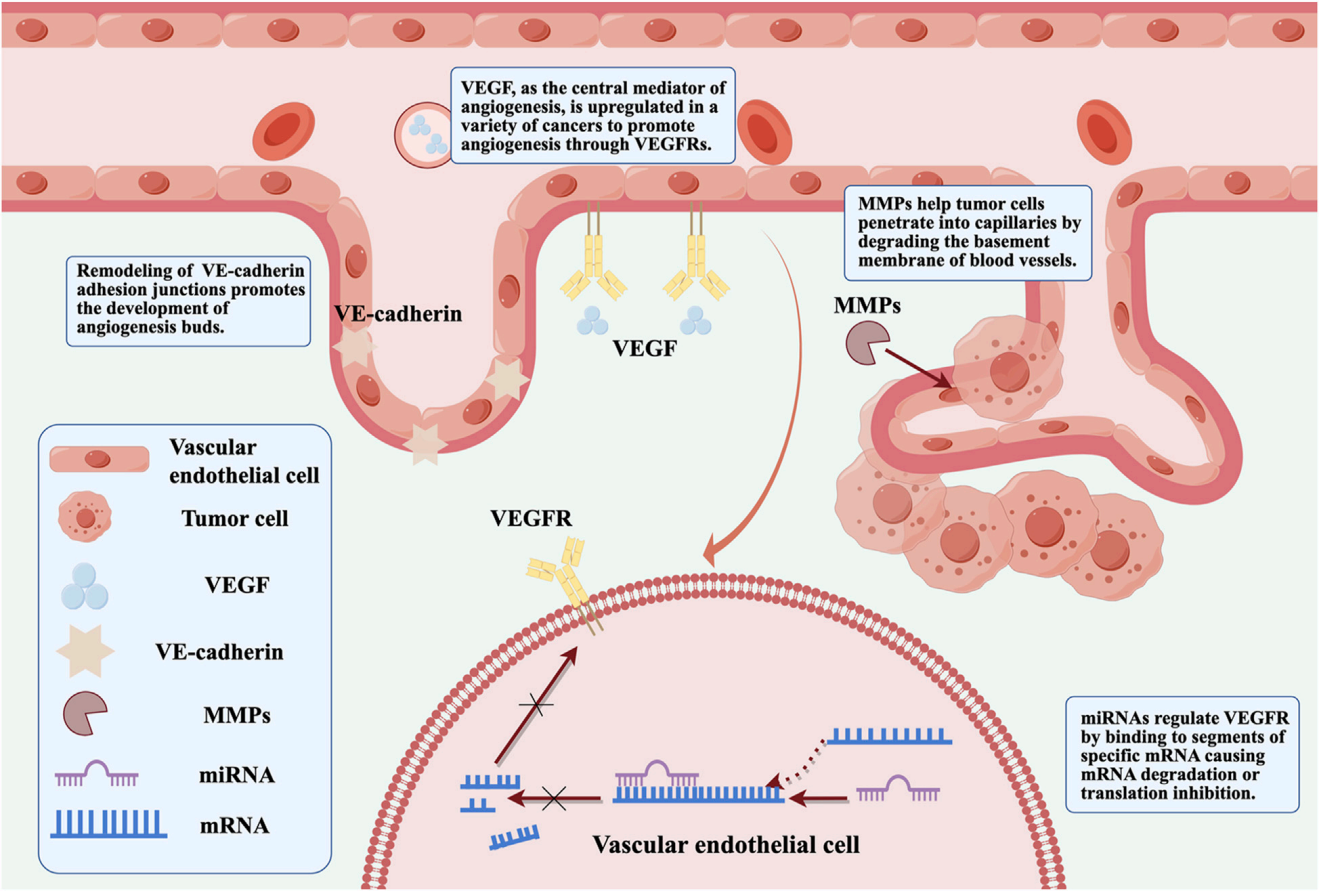
VEGF, MMPs, VE-cadherin and non-coding RNA are involved in regulating the specific mechanisms of tumor angiogenesis (By Figdraw).

The mechanisms underlying tumor angiogenesis and VM formation involve hypoxia, EMT, and activation of tumor-associated fibroblasts and tumor-associated macrophages. Many molecules participate in one or more of these processes that regulate tumor angiogenesis, such as vascular endothelial growth factor (VEGF), MMPs, VE-cadherin, and non-coding RNAs (Geng et al., 2022) that emerged recently (Figure 1). Further, ART inhibits melanoma vasculogenic mimicry by inhibiting the HIF-1α/VEGF/PDGF pathway (Ma et al., 2024b). ART and other artemisinin derivatives have been identified as anti-cancer agents due to their anti-proliferative, anti-angiogenic, and anti-inflammatory properties (Zhong et al., 2021). ART also inhibits STAT3 and Src activations and STAT3 related protein expressions. The upregulated expressions of STAT3 related protein by STAT3, play positive roles in melanoma metastasis through promoting cell invasion and angiogenesis (Berkoz et al., 2021). Currently, anti-angiogenesis targeting VEGFR-2 has been considered as an important strategy for cancer therapy (Lu et al., 2022c). ART inhibits choroidal melanoma cell growth through the STAT3/AKT signaling pathway (Yao et al., 2024b). Furthermore, the synergistic effect of ART and sorafenib (SOR) can inhibit non-Hodgkin lymphoma (NHL) cell viability and have anti-angiogenic activity. Further studies showed that gene inhibition of STAT3 could promote iron apoptosis and cell apoptosis induced by ART/SOR (Chen Y. et al., 2023).

### 3.4 Affecting the tumor microenvironment

The tumor microenvironment refers to the non-cancerous cells and the molecular components produced by the tumor. The interaction between tumor cells and tumor microenvironment plays an important role in tumor genesis, development, metastasis and response to therapy, which has attracted wide attention (Xiao and Yu, 2021). DHA (a metabolite produced in the liver from ART and artemether) regulates the crosstalk between autophagy and IFI16/caspase-1 inflammasome, which inhibits IL-1β production in tumor microenvironment (Shi et al., 2020). Furthermore, nanozyme-like single-atom catalyst combined with ART produces abundant cell cytotoxic radicals in tumor microenvironment (TME) for inhibiting tumor growth (Lv et al., 2023). Monocytes are components of the tumor microenvironment related to cancer progression and immune escape. ART induced changes in the monocyte phenotype are mediated by JAK2/STAT3 downregulation (Mancuso et al., 2021c).

### 3.5 Modulating immune response

ART has an immunomodulatory effect on various immune cells and cytokines of the immune system, also it shows different regulatory effects in different immune states (Lin et al., 2022). Flagella of tumor-targeting bacteria trigger local hemorrhage to reprogram tumor-associated macrophages for improved anti-tumor therapy (Xu et al., 2023). Other related studies report for the first time about the anti-complement bioactivities of ART and suggest a potential therapeutic benefit of ART in complement-related human diseases (Song et al., 2021). ART protects immunosuppression mice induced by glucocorticoids via enhancing pro-inflammatory cytokines release and bacterial clearance, and does indeed demonstrate to possess immunomodulatory effects (Wang et al., 2021). ART suppresses TAZ/PD-L1–induced T-cell growth inhibition *in vitro* and enhances anti-tumor immunity by recruiting infiltrating CD8^+^ T-cells in syngeneic mouse models (Cao et al., 2022). NK cells can eliminate virus-infected cells and tumor cells nonspecifically (Lin et al., 2022) while ART is able to enhance the cytotoxicity of NK92 cells (Zhang J. et al., 2021; Lin et al., 2022).

### 3.6 Overcoming drug resistance in cancer cells

**FIGURE 2:**
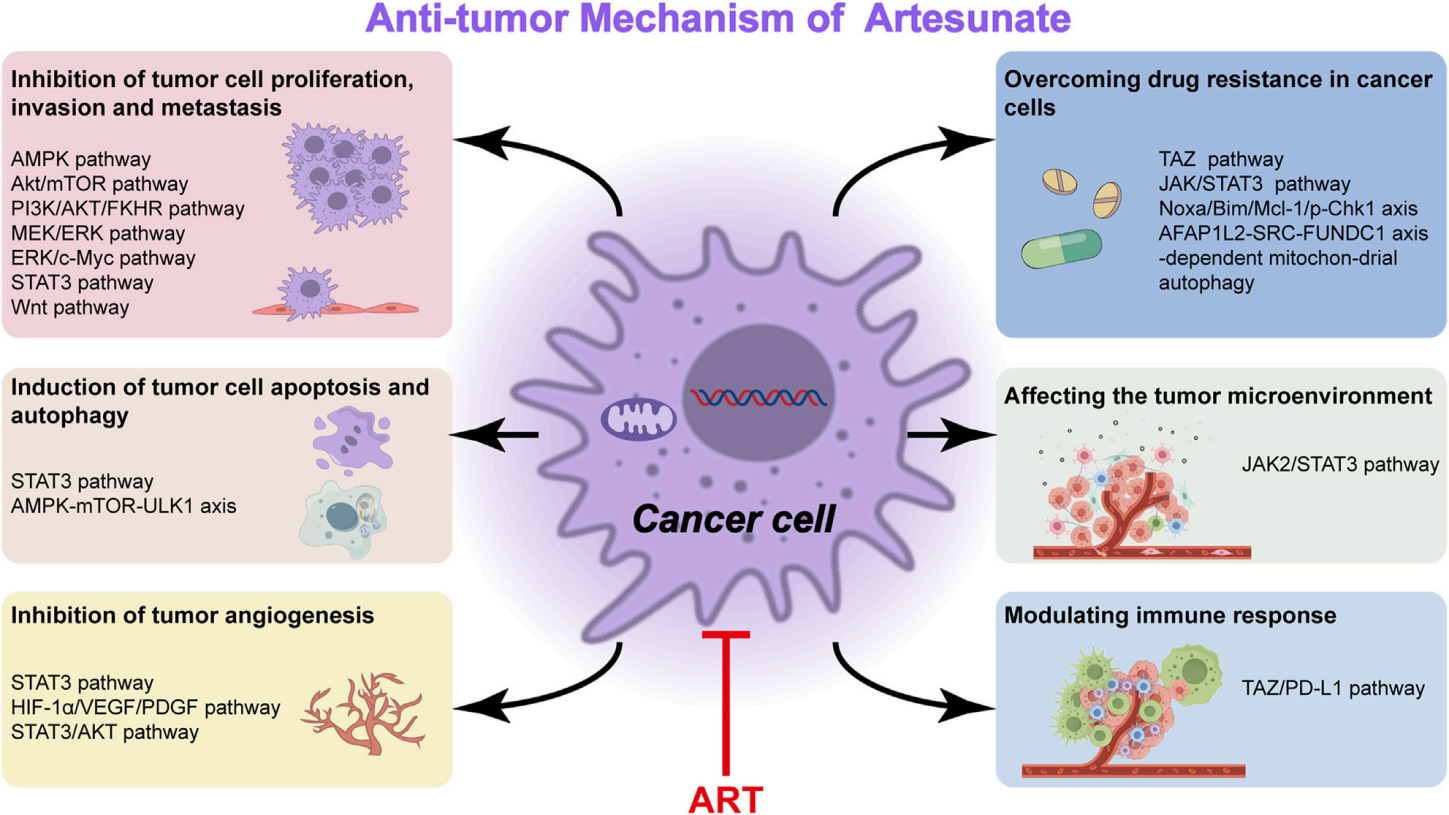
Anti-tumor mechanism of artesunate. Some elements originate from Figdraw.

Overcoming drug resistance and seeking new therapeutic strategies are the main focus of tumor research. Natural products serve as effective substances against drug resistance because of their diverse chemical structures and pharmacological effects. Their main mechanisms for reversing resistance include regulating proteins involved in resistance, targeting non-apoptotic cell death, and inducing other types of non-apoptotic cell death. Signaling pathways associated with tumor resistance include epidermal growth factor receptor (EGFR), renin-angiotensin system (Ras), phosphatidylinositol-3 kinase/protein kinase B (PI3K/Akt), Wnt, Notch, transforming growth factor-β (TGF-β) and their specific natural product signaling pathway inhibitors. This has implications for how to prevent drug resistance to cancer treatment (Yang et al., 2022). Recent evidence shows that lysosomal function is associated with drug resistance of cancer cells. The results suggest that ART or other inhibitors of lysosomal function would be potential in the treatment of cancer cells with drug resistance caused by the enhanced lysosomal function (Li Z. et al., 2021). ART promotes anti-tumor immunity and overcomes EGFR-TKI resistance in non-small-cell lung cancer by enhancing oncogenic TAZ degradation (Cao et al., 2022). In addition, ART is effective against chemoresistant anaplastic thyroid carcinoma by targeting mitochondrial metabolism (Ma and Fei, 2020). Most patients with advanced HCC develop resistance to sorafenib early during treatment. While ART or ginsenoside Rg3 (a main bioactive triterpenoid saponin of red ginseng) in combination with ART can be used to overcome resistance to Sorafenib in hepatocellular carcinoma cells (He et al., 2021; Yao et al., 2022). ART significantly inhibits proliferative and metabolic aspects of cisplatin-sensitive and cisplatin-resistant bladder cancer (BCa) cells, it may hold potential in treating advanced and therapy-resistant BCa (Zhao F. G. et al., 2020). ART reverses the resistance of AML cells to AraC by blocking the JAK/STAT3 signaling pathway, and the combination of ART and cytarabine significantly reduced the proliferation of AML cells and increased its apoptosis rate (Su et al., 2023). ART targeting Noxa/Bim/Mcl-1/p-Chk1 axis improves drug resistance of venetoclax combined with cytarabine in AML, providing a new triple therapy for AML treatment (Zhang J. et al., 2022). ART mitigates sorafenib resistance in hepatocellular carcinoma (HCC) patients by exacerbating AFAP1L2-SRC-FUNDC1 axis-dependent mitochondrial autophagy (Ma Z. et al., 2024) (Figure 2).

## 4 ART against different types of neoplasm

**FIGURE 3:**
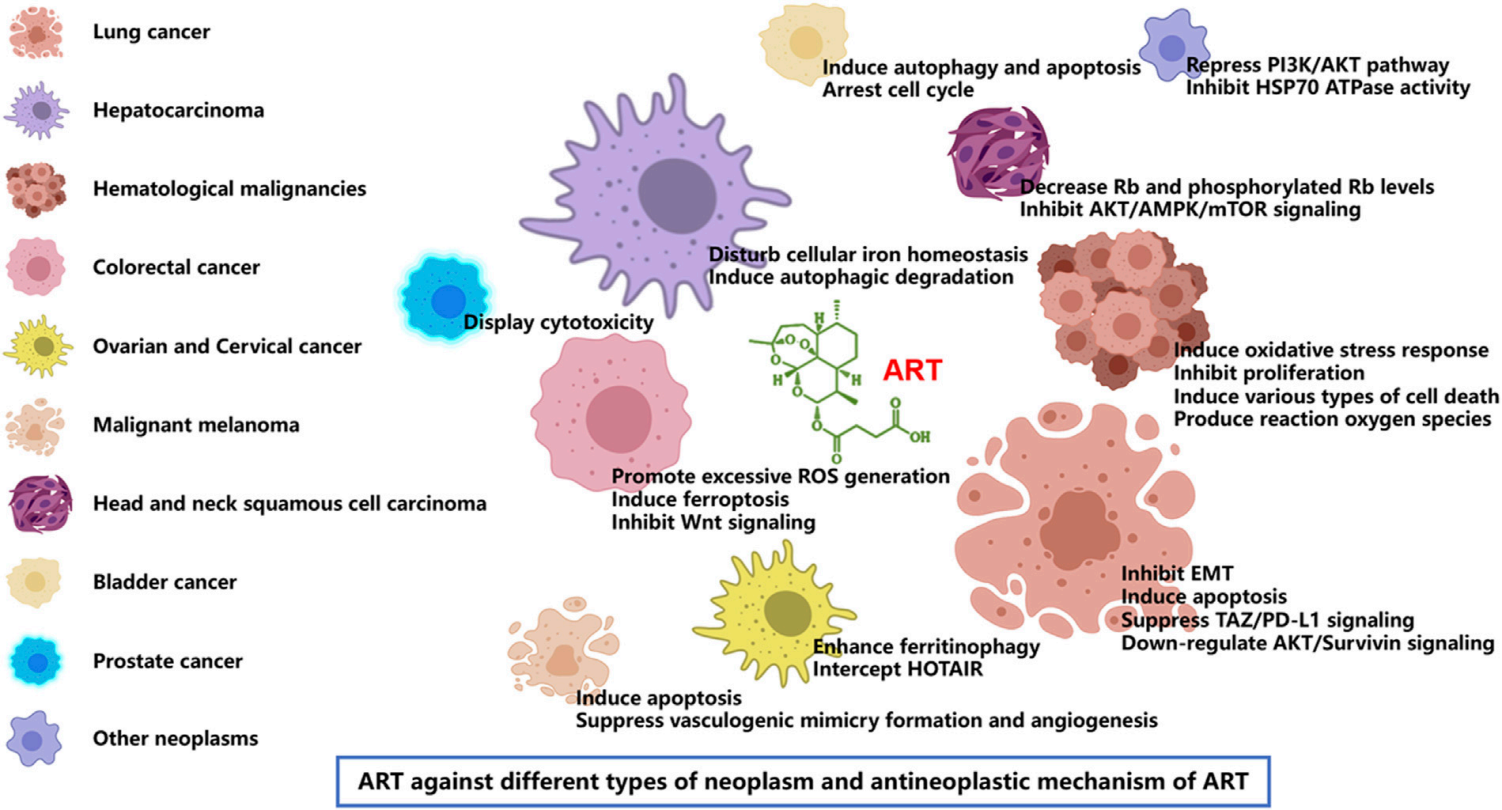
Artesunate inhibits growth and survival of many types of cancer including lung cancer, hepatocarcinoma, hematological malignancies, colorectal cancer, ovarian cancer, cervical cancer, malignant melanoma, head and neck tumor, bladder cancer, and prostate cancer. ART has the same or different mechanisms of action against different tumors, including the inhibition of tumor cell proliferation, migration and invasion [22], induction of tumor cell apoptosis and autophagy [23], regulation of cell signal transduction [24], inhibition of tumor angiogenesis [25] and many other aspects.

**FIGURE 4:**
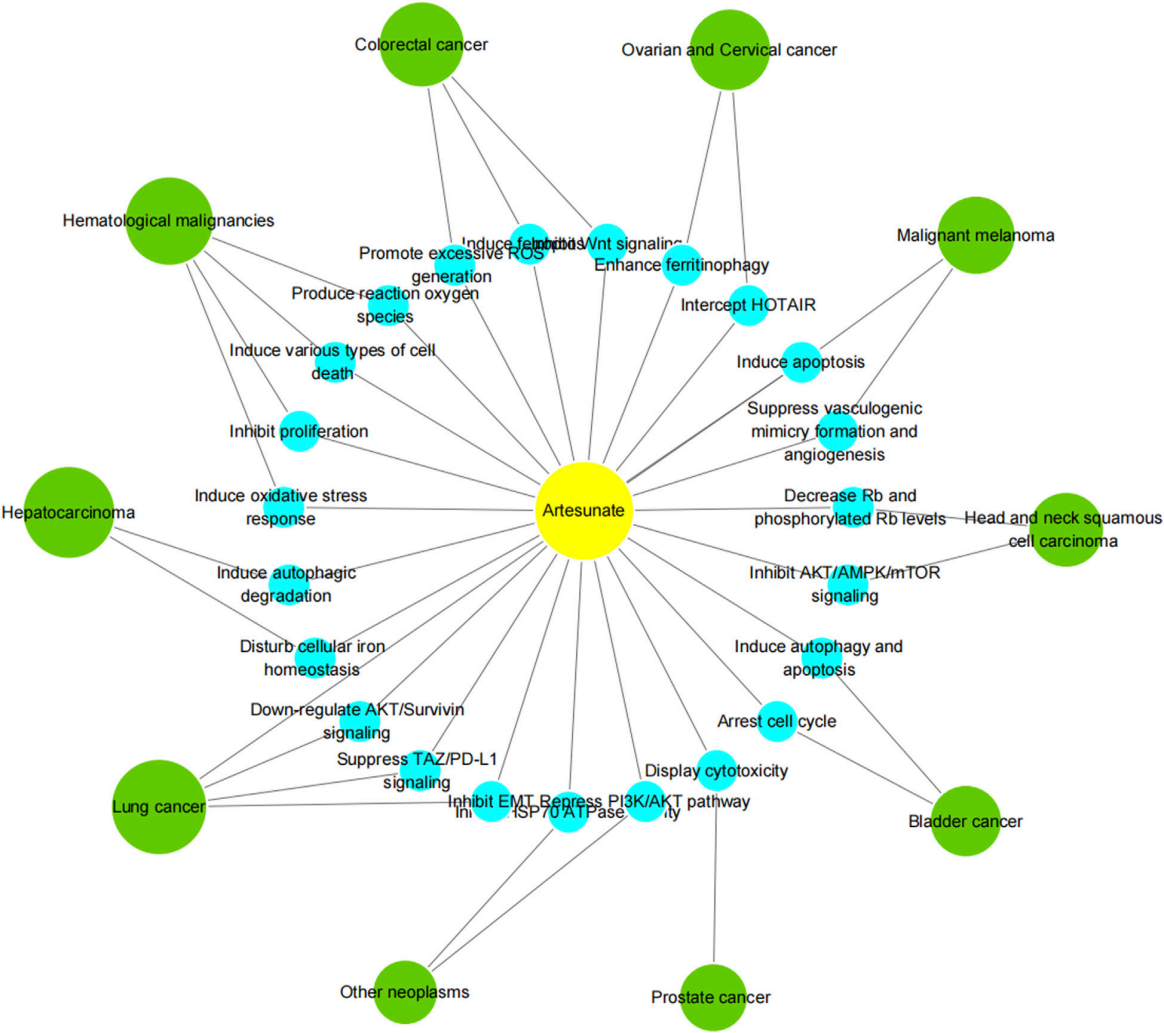
Network of Artesunate’s function in different types of neoplasm.

According to the latest estimates by the International Agency for Research on Cancer (IARC) through 2022, lung cancer was the most common cancer, followed by female breast, colorectal, prostate and stomach cancers. Lung cancer was also the leading cause of cancer death, followed by colorectal, liver, and female breast and stomach cancers. Broken down by sex, breast cancer and lung cancer were the most common cancers in women and men, respectively (Bray et al., 2024). Cancer is a major social, public health and economic problem of the 21st century. In the following, we focus on the role of artesunate in more than a dozen cancers, and present the latest trends and potential prospects for its prevention and control of tumors (Table 3; Figures 3, 4).

**TABLE 3:** Summary of the effects of artesunate on different neoplasms.

Neoplasms	Mechanism of action	References
Lung cancer	Inhibit EMT, regulate mitochondrial membrane potential, induce apoptosis, downregulate the AKT/Survivin Signaling Pathway, suppress TAZ/PD-L1 signaling	Wang et al. (2020a), Zhao et al. (2020c), Cao et al. (2022) Zhang et al. (2022d), Somavarapu et al. (2023)
Hepatocarcinoma	Disturb cellular iron homeostasis, target GBA and induce autophagic degradation	Jiang et al. (2021), Chen et al. (2022)
Lymphoma and Multiple myeloma	Induce oxidative stress response, inhibit proliferation, induce various types of cell death, produce reaction oxygen species, induce differentiation and signal transducersexert, exert anti-ATLL effects, impair STAT3 signaling, target ROS/Bim and TFRC/Fe^2^+ pathways, induces endoplasmic reticulum (ER) stress	Ishikawa et al. (2020), Li et al. (2020), Cao et al. (2021), Mancuso et al. (2021a), Mancuso et al. (2021c), Chen et al. (2023b), Liu et al. (2023a)
Leukemia	Switch monocytes to an inflammatory phenotype, four primary disease targets, CASP3, EGFR, MAPK1, and STAT3	Mancuso et al. (2021c), Tao et al. (2023)
Colorectal cancer	Suppress cellular senescence, promote excessive ROS generation, suppress the expression of survivin, induce ferroptosis, inhibit Wnt signaling	Eitae and Park (2020), Hamoya et al. (2021), Huang et al. (2022b), Xia et al. (2023)
Ovarian cancer	Enhance ferritinophagy	Koike et al. (2022)
Cervical cancer	Intercept HOTAIR	Zhou et al. (2020c)
Malignant melanoma	Induce apoptosis, suppress choroidal melanoma vasculogenic mimicry formation and angiogenesis via the Wnt/CaMKII signaling axis, regulate the AKT/mTOR pathway, regulating the HIF-1α/VEGF/PDGF pathway, down-regulating EFNA3	Berkoz et al. (2021), Geng et al. (2022), Wroblewska-Luczka et al. (2023), Ma et al. (2024b), Yao et al. (2024a), Yao et al. (2024b)
Head and neck squamous cell carcinoma	decrease in Rb and phosphorylated Rb levels, inhibit macrophage migration inhibitory factor, induce mitochondrial dysfunction-dependent oxidative damage, inhibit Akt/AMPK/mTOR signaling	Yu et al.; Xiao et al. (2020), Okamoto et al. (2023)
Bladder cancer	Inhibit the viability, proliferation and migration of BCa cells, induce autophagy	Zhou et al. (2020b), Chen et al. (2023a)
Prostate cancer	Display cytotoxicity	Adedeji et al. (2020), Fabian et al. (2022)
Cutaneous squamous cell carcinoma	Repress PI3K/AKT pathway	Huang et al. (2022a)
Breast cancer	Inhibit HSP70 ATPase activity	Pirali et al. (2020), Svolacchia et al. (2023)
Esophageal cancer	Target HK1	Jin et al. (2022)
Renal cell carcinoma	Arrest cell cycle, induce ferroptosis	Markowitsch et al. (2020)
Thyroid carcinoma	Target mitochondrial metabolism, inhibit the PI3K/AKT/FKHR signaling pathway	Ma and Fei (2020), Xu et al. (2022)
Prolactinoma	Inhibit mitochondrial metabolism, Induce apoptosis	Zhang et al. (2020b)
Glioblastoma	Induce ferroptosis via modulation of p38 and ERK signaling pathway	Song et al. (2022)

### 4.1 Lung cancer

Lung cancer is the main cause of cancer-related deaths around the world (Sherry, 2022). Recent studies have found that ART has significant therapeutic potential in multiple respiratory diseases (Zhang et al., 2022b). Lung cancer, the most common malignant tumor, is classified into NSCLC and small cell lung cancer (SCLC). ART was involved in inhibiting the epidermal interstitial transformation (EMT) of NSCLC cells by up-regulating the expression of epithelial marker E-cadherin and inhibiting the protein and mRNA levels of mesenchymal markers N-cadherin, vimentin and FN1 in A549 and H1975 cells (Wang J. S. et al., 2020). ART suppresses lung cancer cells growth by regulating mitochondrial membrane potential, inducing apoptosis (Zhao et al., 2020c) and down-regulating the AKT/Survivin Signaling Pathway (Zhang W. W. et al., 2022). In addition, ART enhances anti-tumor immunity and overcomes EGFR-TKI resistance in NSCLC at least in part by suppressing TAZ/PD-L1 signaling (Cao et al., 2022). Results have demonstrated that the ART-loaded PLGA porous microsphere could achieve excellent anti-cancer efficacy, providing a potential approach for NSCLC treatment via the pulmonary administration (Xiong et al., 2021). In addition, spray-dried micelles containing artesunate have the potential to be used as a dry powder formulation for inhalation in the treatment of lung cancer (Somavarapu et al., 2023).

### 4.2 Hepatocarcinoma

Hepatocellular carcinoma (HCC), one of the most common cancers, causes the fourth cancer-related deaths worldwide (Wang M. Y. et al., 2020). An effective strategy for HCC therapy is the perturbation of iron metabolism. ART regulates the unstable iron pool (LIP) and effectively induces ROS-dependent cell death in a variety of HCC cells, showing anticancer activity (Jiang et al., 2021). Glucosylceramidase (GBA) is required for autophagic degradation, and a preclinical study identified GBA as one of the direct targets of ART, which may have promising potential to inhibit lysosomal autophagy for HCC therapy (Chen et al., 2022). Another research shows that ART alone inhibits the proliferation of five HCC cell lines in a dose-dependent manner, and sorafenib combined with ART exert a synergistic anti-proliferation effect and induced synergistic apoptosis in HCC cell lines (Yao et al., 2020). The synergistic effects include ferroptosis induction (Li Z. J. et al., 2021), apoptosis induction, cell migration inhibition and anti-angiogenesis activity.

### 4.3 Lymphoma, multiple myeloma and leukemia

ARS and its derivatives (ART, DHA, artemether) act in a multi-specific manner against hematological malignancies, for example, lymphoma, multiple myeloma, and leukemia. The main mechanism of ARTs against different types of hematological malignancies comprises oxidative stress response, inhibition of proliferation, induction of various types of cell death (apoptosis, autophagy, ferroptosis, inhibition of angiogenesis), production of reaction oxygen species, induction of differentiation, and signal transducers, amongst others (Li et al., 2020; Mancuso et al., 2021b). ART is effective against adult T-cell leukemia/lymphoma by blocking G1 and/or G2/M phases, reducing the expression of cyclin-dependent kinases 1/2/4/6, cyclin B1/D2/E, and c-Myc, and increasing the expression of p21 (Ishikawa et al., 2020). ART exhibits cytotoxic effects in primary effusion lymphoma (Ishikawa and Mori, 2021) and induces apoptosis, autophagy and ferroptosis in diffuse large B cell lymphoma cells by impairing STAT3 signaling (Chen et al., 2021; Chen et al., 2023a). Beyond that, ART combined with arsenite inhibits the growth of multiple myeloma cells through the PI3K/AKT signaling pathway (Hu G.-F. et al., 2021). Leukemia is a hematological malignancy originated from the bone marrow (Ng et al., 2021). ART induces apoptosis and inhibits the proliferation, stemness, and tumorigenesis of leukemia (Chen et al., 2020). ART converts monocytes, a component of the tumor microenvironment associated with cancer progression and immune escape, into an inflammatory phenotype with the ability to kill leukemia cells (Mancuso et al., 2021c). Also, artesunate has significant anti-leukemia effects in mice by targeting ROS/Bim and TFRC/Fe^2^+ pathways (Liu et al., 2023). ART induces endoplasmic reticulum (ER) stress in leukemia cells eventually led to apoptosis (Mancuso et al., 2021a). Venetoclax plus cytarabine therapy is approved for elderly AML patients. Furthermore, ART induces synergistic apoptosis with venetoclax/cytarabine/daunorubicin accompanied (Li J.-Y. et al., 2022; Zhang et al., 2022b). Molecular docking findings reveal that artesunate is critically important in the therapy of AML due to its high affinity for the four primary disease targets, CASP3, EGFR, MAPK1, and STAT3 (Tao et al., 2023). Besides, ART induces cell death in pediatric AML cell lines through cytochrome c (Hill et al., 2023).

### 4.4 Colorectal cancer

Colorectal cancer has the second highest incidence of malignant tumors and is the fourth leading cause of cancer deaths in China (Cai et al., 2021). The combination of Arte and 5-FU significantly reduced the viability of cancer cells *in vitro* and synergistically inhibited the growth of CRC xenografts *in vivo* (Xia et al., 2023). ART has been reported to be a promising candidate for CRC treatment, which induces senescence and autophagy to inhibit cell proliferation in colorectal cancer by promoting excessive ROS generation (Huang Z. Y. et al., 2022). The study proposes that ART induces cytotoxicity in HCT116 colon cancer cells by suppressing the expression of survivin and partially by ferroptosis (Eitae and Park, 2020). Abnormal Wnt signaling pathways are known to lead to unexpected β-catenin nuclear translocation and promote T-cell factor/lymphoid enhancer factor (TCF/LEF) transcriptional activity, resulting in CRC progression. ART inhibits intestinal tumorigenesis through inhibiting Wnt signaling (Hamoya et al., 2021).

### 4.5 Ovarian cancer and cervical cancer

Ovarian cancer and cervical cancer are life-threatening diseases with a high mortality rate among women (Caird et al., 2022; Yuan et al., 2022). ART has preclinical activity in ovarian cancer that merits further investigation to treat ovarian cancer (McDowell et al., 2021). Enhancement of ferritinophagy is an important step involved in the mechanism of artesunate-induced ferroptosis, and ferritin heavy chain levels may contribute to the regulation of sensitivity in artesunate-induced ferroptosis in ovarian serous carcinoma cells (Koike et al., 2022). Synergetic delivery of ART and isosorbide 5-mononitrate with reduction-sensitive polymer nanoparticles for ovarian cancer chemotherapy (Li et al., 2022b). HOX transcript antisense RNA (HOTAIR), a trans-acting long non-coding RNA (lncRNA), plays an oncogenic role in cervical cancer by promoting cell proliferation, migration, invasion and autophagy, etc. Moreover, the blockade of HOTAIR by ART or propofol shows promise for further development of this lncRNA as a potential therapeutic target in cervical cancer (Zhou et al., 2020a). ART has a cytotoxic effect on squamous cells transformed by HPV. Self-administered vaginal ART inserts are safe and well-tolerated, which can be used at clinically effective doses to treat cervical intraepithelial neoplasia 2/3 (CIN2/3) (Trimble et al., 2020).

### 4.6 Malignant melanoma

Malignant melanoma is a malignant neoplasm of the skin and mucosal tissues (Zahir et al., 2021) characterized by a potential metastatic tumor of melanocytic origin. ART inhibits cellular proliferation of cancer cells by induction of apoptosis (Wroblewska-Luczka et al., 2023). The treatment shows decreased cellular migration, invasion, and colony formation in melanoma cells (Berkoz et al., 2021). ART suppresses choroidal melanoma (CM) vasculogenic mimicry formation and angiogenesis, while angiogenesis and VM are considered to be the main processes to ensure tumor blood supply during the proliferation and metastasis of CM (Geng et al., 2022). In uveal melanoma, ART elevates the apoptotic rate and suppresses C918 cell viability (Jiu et al., 2021). In addition, ART regulates the AKT/mTOR pathway by reducing the expression of Ang-1 in CM cells, thereby inhibiting the occurrence of CM tumors (Yao et al., 2024a). EphrinA3 (EFNA3) promotes CM cell growth and migration by activating the STAT3/AKT signaling pathway, while ART inhibits this process by down-regulating EFNA3 (Yao et al., 2024a). Meanwhile, ART has played a role in suppressing choroidal melanoma by regulating the HIF-1α/VEGF/PDGF pathway (Ma et al., 2024b).

### 4.7 Head and neck squamous cell carcinoma

Artesunate and cisplatin synergistically inhibit HNSCC cell growth and promote apoptosis with artesunate-induced decreases in Rb and phosphorylated Rb levels (Okamoto et al., 2023). Oral squamous cell carcinoma, the most common type of oral cancer, affects more than 275,000 people per year worldwide (Pena-Oyarzun et al., 2020). ARS suppresses tumor growth and induces vascular normalization in oral squamous cell carcinoma via inhibition of macrophage migration inhibitory factor (Ding et al., 2019; Yu et al., 2024). Mitochondrial metabolism has recently gained attention as a promising therapeutic strategy in cancer. ART targets oral tongue squamous cell carcinoma via mitochondrial dysfunction-dependent oxidative damage and Akt/AMPK/mTOR inhibition (Xiao et al., 2020).

### 4.8 Bladder cancer

Urinary bladder cancer is a common urological cancer (Tse et al., 2021) and is the 10th most common cancer type in the world (Bilim et al., 2022). ART can inhibit the viability, proliferation and migration of bladder cancer cells, as well as induce autophagy in a time and dose dependent manner. In addition, ART induced apoptosis of bladder cancer cells by up-regulating ROS and activating AMPK-mTOR-ULK1 pathway (Zhou X. J. et al., 2020). ART impairs growth in cisplatin-resistant bladder cancer cells by cell cycle arrest, apoptosis and autophagy induction, which may hold potential in treating advanced and therapy-resistant bladder cancer (Zhao F. G. et al., 2020). ART induces G2/M cell cycle arrest in HT 1376 and BFTC 909 cell lines, thereby inducing apoptosis and REDOX imbalance, and may be a candidate drug for the treatment of bladder cancer in concert with cisplatin (Chen S. Y. et al., 2023). A new artesunate-metformin dimer triazine derivative AM2 is designed and synthesized by coupling artesunate with metformin. AM2 inhibits the growth of bladder cancer cells T24 by inhibiting cellular adipogenesis associated with the Clusterin/SREBP1/FASN signaling pathway (Lin et al., 2024).

### 4.9 Prostate cancer

Prostate cancer (PC), a malignant tumor occurring in the male prostate, is the second leading cause of cancer-related deaths in men. The lesions have the characteristics of small size and blurry outline (Qian Y. J. et al., 2021). Combining ART with PTX displays cytotoxicity regardless of the type of prostate cancer cell line. This may offer a promising new therapeutic option for the treatment of metastatic hormone-refractory PC (Adedeji et al., 2020; Fabian et al., 2022). ART induced apoptosis of parent and DX-resistant DU145 cells by increasing ROS, indicating that ART inhibited the growth of docetaxel-resistant PC cells (Vakhrusheva et al., 2022).

### 4.10 Other neoplasms

ART evidently attenuates the migration, invasion and proliferation, lessened cell numbers at G2/M phase and triggers apoptosis of CSCC cells, which may be intensely related to the PI3K/AKT pathway repression (Huang X. Y. et al., 2022). ART induces the death of breast cancer cell lines 4T1 and MCF-7 by inhibiting the expression of HSP70 and Bcl-2 (Pirali et al., 2020; Svolacchia et al., 2023). ART targets HK1, promotes the degradation of HK1, reduces the expression of HIF-1α and PKM2, which are key glycolytic enzymes, and plays an anti-EC role (Jin et al., 2022). Furthermore, ART can reverse gemcitabine (dFdC) resistance in combination with dFdC in dFdC-resistant Panc-1 cells *in vitro* (Yao et al., 2022b). ART therapy significantly increases the cytotoxicity of Sunitinib-resistant RCC cells and inhibited proliferation and clonal growth. ART inhibits the growth of KAKI-1, 786-O, and A-498 cell lines through G0/G1 phase arrest and significant regulation of cell cycle regulators, and inhibits the growth of KTCTL-26 through ROS production, ferroptosis, and metabolism (Markowitsch et al., 2020). The inhibition of ART on chemotherapy-sensitive (8505C and KAT-4) and drug-resistant (8505C-R and KAT-4-R) ATC cells is effective against chemotherapy-resistant anaplastic thyroid cancer by inhibiting mitochondrial function, leading to oxidative stress and damage (Ma and Fei, 2020). ART inhibits apoptosis, proliferation and migration of thyroid cancer cells by inhibiting the PI3K/AKT/FKHR signaling pathway (Xu et al., 2022). ART exerts anti-prolactinoma activity by inducing G0/G1 phase arrest and cell apoptosis, thereby inhibiting mitochondrial metabolism and inducing cell apoptosis (Zhang W. Y. et al., 2020). ART influences iron apoptosis by regulating iron homeostasis and p38 and ERK signaling pathways. These findings support the role of ART in inducing ferroptosis through this pathway in glioblastoma (Song et al., 2022). In breast cancer, artesunate also induces apoptosis in cancer cells (Wen et al., 2024).

## 5 Clinical evidence of ART as an anti-cancer therapy

At present, artesunate has been gradually used in clinical studies to treat tumors, and has become a promising strategy for the treatment of cancer. In glioblastoma cells, artesunate acts as a supplement for cancer treatment. Clinical trials have shown that artesunate itself is cytotoxic and enhances the cytotoxicity of temozolomide, and therefore has the potential to enhance the therapeutic efficacy of glioblastoma (Strik et al., 2024). Artesunate is effective, safe and well tolerated for the treatment of cervical intraepithelial neoplasia 2/3 (CIN2/3) (Trimble et al., 2020). Phase I clinical trials evaluated artesunate suppositories as effective in treating HPV-infected cells with cytotoxic effects while having minimal effects on healthy cells (Fang et al., 2023). Artesunate has shown anticancer activity both *in vitro* and *in vivo* against hematological malignancies in a multispecific manner. The main mechanisms of its action on leukemia, multiple myeloma and lymphoma cells include oxidative stress response, inhibition of proliferation and induction of various types of cell death (Li et al., 2020; Mancuso et al., 2021b). Because artesunate drugs are highly effective and well tolerated without side effects, they could be applied in the future as anti-tumor therapy alone or in combination with standard chemotherapy after further clinical trials in various tumors are completed.

## 6 Conclusion and perspectives

Cancer is one of the most important and common public health problems on Earth endangering human health. And its incidence rates continue to rise. As anti-tumor drugs have always been the most common methods for treating cancers, searching for new anti-tumor agents is of great significance (Sekeroglu and Tuncal, 2021; He et al., 2023). The use of herbal products is booming all over the world because of being believed as safer than conventional drugs and free of side effects (Mwankuna et al., 2023). Chinese herbal medicine (CHM) has long been applied in the clinic due to its advantages of low toxicity and polypharmacology (Li et al., 2022). CHM plays a positive role in regulating patients' immune system, which helps cancer patients to fight against cancer itself and finally improves patients' life quality (Wang S. M. et al., 2020). ART, a sesquiterpene lactone endoperoxide isolated from Chinese herbal medicine, displays excellent anti-tumor and anti-inflammatory activity (Lu W. J. et al., 2022; Wang et al., 2022). It is safe, efficacious and well-tolerated anti-malarial (Savargaonkar et al., 2020). ART possesses profound cytotoxic activity against tumor cells (Khanal, 2021), which brings new hope for the treatment of diseases. The combination of ART and other anti-tumor drugs may provide a new idea for the treatment of tumors in the future (Duarte and Vale, 2020). In this paper, the anti-tumor mechanism of artesunate and its corresponding signaling pathway are reviewed, and the anti-tumor mechanism of artesunate in different types of tumors is analyzed and explained. In addition to this, we also provide a separate explanation of the anti-tumor mechanism of artesunate in different types of tumors. It provides a comprehensive reference for further study of the anti-tumor mechanism of artesunate, and is conducive to expanding its clinical application. In conclusion, the efficacy of ART as an anticancer agent has been demonstrated in multiple tumor types. However, the anti-tumor mechanism of ART is not completely clear, so it needs to be further studied to obtain more theoretical support and experimental basis.
