# Supplementary figures and images for: Anti-tumor mechanism of artesunate
Source: Front Pharmacol. 2024 Oct 25;15:1483049. doi: 10.3389/fphar.2024.1483049 (PMC11549674; doi:10.3389/fphar.2024.1483049)

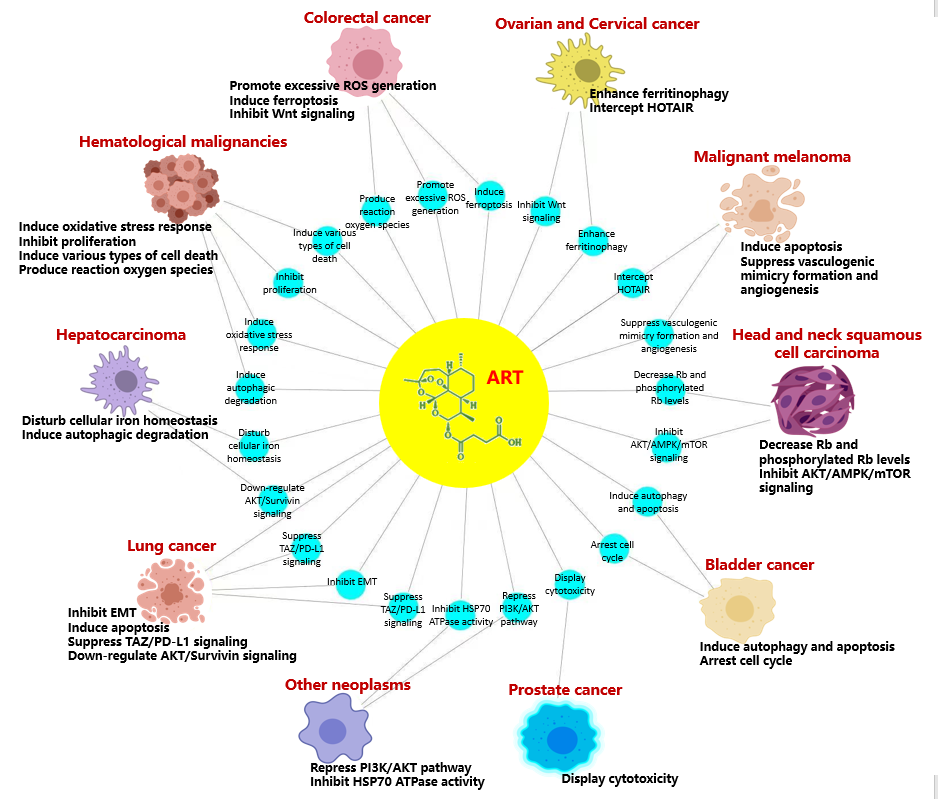

Supplement: Supplementary file 1 [file Image1.TIF]
